# Epidemiological profile and clinical outcomes of patients with mucormycosis: the multicenter retromucor study from Türkiye (2004–2024)

**DOI:** 10.1007/s10096-025-05397-x

**Published:** 2026-01-23

**Authors:** Yeliz Çiçek, Rıdvan Dumlu, Mehmet Koçak, Betül Zehra Pirdal, Mehmet Çelik, Neşe Saltoğlu, Recep Tekin, Süheyla Kömür, Seyit Ali Büyüktuna, Ayşe Batırel, Şua Sümer, Nazlım Aktuğ Demir, Zehra Çağla Karakoç, Duygu Çerçioğlu Özdemir, Ayşe Turunç Özdemir, Ayşin Kılınç Toker, Şafak Kaya, Buket Ertürk Şengel, Nagehan Didem Sarı, Handan Alay, Fatma Kesmez Can, Fatma Bayrak Erdem, Asiye Yir, Türkkan Öztürk Kaygusuz, Gülten Ünlü, Özlem Güler, Nadide Ergün, Emine Günal, Selin Özdemir, Emre Bayhan, Alper Tahmaz, Eyüp Arslan, Nur Bahar Oğuz, Aybike Özdağ, Didem Akal Taşcıoğlu, Ece Demirkırkan, Tuba Turunç, Ebru Oruç, Gamze Kalın Ünüvar, Ali Mert, KLİMİK Fungal Study Group

**Affiliations:** 1 Department of Infectious Diseases and Clinical Microbiology, Faculty of Medicine, Istanbul Medipol University, İstanbul, Türkiye; 2 Epidemiology Doctorate Program, Graduate School of Health Sciences, Istanbul Medipol University, İstanbul, Türkiye; 3 Department of Biostatistics and Medical Informatics, Istanbul Medipol University, İstanbul, Türkiye; 4 General Directorate of Health Promotion, Ministry of Health of Türkiye, Ankara, Türkiye; 5 Department of Infectious Diseases and Clinical Microbiology, Harran University Faculty of Medicine, Şanlıurfa, Türkiye; 6 Cerrahpaşa Faculty of Medicine, Department of Infectious Diseases and Clinical Microbiology, Istanbul University-Cerrahpaşa, İstanbul, Türkiye; 7 Faculty of Medicine, Department of Infectious Diseases and Clinical Microbiology, Dicle University, Diyarbakır, Türkiye; 8 Faculty of Medicine, Department of Infectious Diseases and Clinical Microbiology, Balcalı Hospital, Çukurova University, Adana, Türkiye; 9 Faculty of Medicine, Department of Infectious Diseases and Clinical Microbiology, Sivas Cumhuriyet University, Sivas, Türkiye; 10 Department of Infectious Diseases and Clinical Microbiology, SBÜ Kartal Dr. Lütfi Kırdar City Hospital, İstanbul, Türkiye; 11 Faculty of Medicine, Department of Infectious Diseases and Clinical Microbiology, Selçuk University, Konya, Türkiye; 12 Faculty of Medicine, Department of Infectious Diseases and Clinical Microbiology, İstinye University, İstanbul, Türkiye; 13 Department of Infectious Diseases and Clinical Microbiology, Kayseri City Hospital, Kayseri, Türkiye; 14 Department of Infectious Diseases and Clinical Microbiology, SBÜ Gazi Yaşargil Training and Research Hospital, Diyarbakir, Türkiye; 15 Faculty of Medicine, Department of Infectious Diseases and Clinical Microbiology, Marmara University, Istanbul, Türkiye; 16 Department of Infectious Diseases and Clinical Microbiology, SBÜ Istanbul Training and Research Hospital, Istanbul, Türkiye; 17 Faculty of Medicine, Department of Infectious Diseases and Clinical Microbiology, Atatürk University, Erzurum, Türkiye; 18 Department of Infectious Diseases and Clinical Microbiology, Bakırköy Dr. Sadi Konuk Training and Research Hospital, Istanbul, Türkiye; 19 Department of Infectious Diseases and Clinical Microbiology, Başaksehir Cam and Sakura City Hospital, Istanbul, Türkiye; 20 Faculty of Medicine, Department of Infectious Diseases and Clinical Microbiology, Firat University, Elazig, Türkiye; 21 Department of Infectious Diseases and Clinical Microbiology, Kocaeli City Hospital, Kocaeli, Türkiye; 22 Faculty of Medicine, Department of Infectious Diseases and Clinical Microbiology, Kocaeli University, Kocaeli, Türkiye; 23 Department of Infectious Diseases and Clinical Microbiology, Izmir City Hospital, Izmir, Türkiye; 24 Department of Infectious Diseases and Clinical Microbiology, Prof. Dr. Cemil Taşcıoğlu City Hospital, Istanbul, Türkiye; 25 Department of Infectious Diseases and Clinical Microbiology, Izmir Bozyaka Training and Research Hospital, Izmir, Türkiye; 26 Department of Infectious Diseases and Clinical Microbiology, Adiyaman Training and Research Hospital, Adiyaman, Türkiye; 27 Department of Infectious Diseases and Clinical Microbiology, Antalya Training and Research Hospital, Antalya, Türkiye; 28 Department of Infectious Diseases and Clinical Microbiology, SBÜ Sancaktepe Sehit Prof. Dr. Ilhan Varank Training and Research Hospital, Istanbul, Türkiye; 29 Department of Infectious Diseases and Clinical Microbiology, SBÜ Adana City Training and Research Hospital, Adana, Türkiye; 30 Faculty of Medicine, Department of Infectious Diseases and Clinical Microbiology, Erciyes University, Kayseri, Türkiye; 31 Faculty of Medicine, Department of Internal Medicine, Istanbul Medipol University, Istanbul, Türkiye

**Keywords:** Mucormycosis, Epidemiology, Diabetes mellitus, Amphotericin b, Immunosuppression, Prognosis

## Abstract

**Purpose:**

Mucormycosis is a rapidly progressive angioinvasive fungal infection with limited large-scale outcome data. We aimed to describe the epidemiology, clinical spectrum, treatment strategies, and predictors of outcomes in adult patients with proven mucormycosis in Türkiye.

**Methods:**

We conducted a nationwide, retrospective study of 280 adults diagnosed with proven mucormycosis between 2004 and 2024 across 27 tertiary centers. Case definitions were applied according to the European Organization for Research and Treatment of Cancer and the Mycoses Study Group Education and Research Consortium. Clinical characteristics, antifungal management, surgical interventions, and outcomes were systematically analyzed.

**Results:**

The median age of the cohort was 60 years, and diabetes mellitus, frequently complicated by ketoacidosis, was the most common underlying condition. Rhino-orbital-cerebral disease represented the predominant clinical form, whereas pulmonary, gastrointestinal, and disseminated presentations were less frequent but carried less favorable prognoses. Liposomal amphotericin B was the main first-line therapy, often combined with surgery, although antifungal treatment was initiated at a median of five days after symptom onset. Ninety-day mortality was 42.1% by Kaplan–Meier estimation (crude 49.3%) and reached 49.3% (crude 55.0%) at 365 days. Crude mortality was lowest in patients who did not undergo surgery; however, this association was not significant in the time-dependent Cox regression analysis, suggesting confounding by baseline severity. Poor outcomes were associated with corticosteroid exposure, prolonged neutropenia, and neurological manifestations, while a history of sinusitis appeared to be associated with improved survival.

**Conclusion:**

In this multicenter study, survival was mainly determined by host-related factors rather than the choice of antifungal agent. Corticosteroid exposure, prolonged neutropenia, and neurological involvement independently predicted poor outcomes, whereas a prior history of sinusitis was linked to improved survival, likely reflecting earlier diagnosis and intervention. These findings reinforce the need for heightened clinical awareness, rapid recognition, and prompt multidisciplinary management to improve outcomes in patients with mucormycosis.

**Supplementary Information:**

The online version contains supplementary material available at 10.1007/s10096-025-05397-x.

## Introduction

Mucormycosis is an angioinvasive infection caused by filamentous fungi of the order Mucorales. It is characterized by vascular thrombosis, tissue infarction, and rapid clinical deterioration in the absence of prompt recognition and treatment [1]. Reliable global surveillance data remain limited; however, available estimates suggest an incidence ranging from 0.005 to 1.7 cases per million population annually [2]. The burden varies considerably across regions, with rates as high as 140 per million in India, compared with approximately 0.35 per million in the United States and 0.4 to 1.2 per million in Europe [3, 4].

Clinical manifestations depend on the route of fungal entry and the host immune status [5]. Rhino-orbital-cerebral (ROCM) disease is the most frequent presentation, typically manifesting as acute sinusitis with facial pain and nasal congestion, and it may rapidly extend to the orbit and central nervous system, leading to altered mental status or focal neurological deficits [1]. Pulmonary mucormycosis usually occurs in neutropenic or mechanically ventilated patients and is characterized by fever, nonproductive cough, hemoptysis, and radiographic findings such as nodules, consolidation, or cavitation [6]. Cutaneous infection usually results from direct inoculation after trauma or burns, progressing from erythema and induration to necrotic ulcers characterized by black eschar that may involve subcutaneous tissue, muscle, and bone [5]. Gastrointestinal disease, although rare, presents with abdominal pain, bleeding, or perforation, particularly in malnourished infants and critically ill adults [7, 8]. Disseminated infection, defined as the involvement of two or more noncontiguous sites, is uncommon but associated with exceedingly high mortality without rapid intervention [1].

Predisposing factors include conditions that impair innate immunity or promote fungal proliferation. Poorly controlled Diabetes mellitus (DM) particularly when complicated by ketoacidosis remains the predominant risk factor worldwide as hyperglycemia and acidosis impair neutrophil chemotaxis and phagocytosis while acid mediated iron release fosters Mucorales growth [1, 9]. In Western populations hematological malignancies such as acute myeloid leukemia and hematopoietic stem cell transplantation (HSCT) account for 30 to 40% of cases driven by prolonged neutropenia and high dose corticosteroid regimens [3]. Solid organ transplant (SOT) recipients face elevated incidence of 0.3 to 2% with additional contributors including deferoxamine therapy, prolonged corticosteroids, dialysis dependent renal failure, severe burns or trauma and intensive care interventions [10].

Timely initiation of antifungal therapy combined with surgical intervention is crucial for improving survival. The European Confederation of Medical Mycology (ECMM) recommends liposomal amphotericin B at 5–10 mg/kg daily, with an initial 10 mg/kg dose in cases of central nervous system involvement [10]. Combination regimens pairing polyenes with azoles or echinocandins such as caspofungin have demonstrated limited benefit in refractory cases [11]. Once clinical stabilization is achieved, patients may transition to oral step-down therapy with either posaconazole (300 mg daily) or isavuconazole, initiated with six loading doses of 372 mg every eight hours followed by 372 mg daily, guided by therapeutic drug monitoring and drug–drug interaction profiles [10, 12]. Emerging modalities, including fosmanogepix [13] and adjunctive immunomodulators such as interferon-γ and GM-CSF [9], are currently under investigation.

Early surgical debridement within 48–72 h of diagnosis has been consistently associated with improved survival [1]. Surgical approaches vary according to disease localization, ranging from endoscopic sinus clearance with orbital exenteration when necessary, to pulmonary resection for localized lesions [10]. Cutaneous cases, particularly in the context of extensive trauma or burns, often require radical excision of necrotic tissue, frequently necessitating staged procedures [5].

Mortality among patients with mucormycosis remains unacceptably high [10]. Recent multicenter studies report 90-day mortality rates of 50–60% and a median survival of less than two months after diagnosis [4]. The poorest outcomes are observed in severe disease forms: gastrointestinal mucormycosis carries mortality rates exceeding 85% [7], whereas disseminated disease approaches 100% [9]. In ROCM, prompt combined therapy can reduce 90-day mortality to approximately 45%; however, survivors often experience substantial sequelae, including vision loss, cranial nerve deficits, and cognitive impairment [5]. Long-term survival beyond one year is poorly characterized, but available data suggest a persistent risk of relapse, chronic complications, and reduced quality of life [9]. Major predictors of poor outcome include diagnostic delay, renal failure, disseminated infection, and absence of surgical intervention [10].

National and international data on the epidemiology and long-term outcomes of mucormycosis remain limited. This study assembled a multicenter cohort from Türkiye, including adults with proven mucormycosis diagnosed between 2004 and 2024. The primary objective was to identify demographic and clinical predictors of all-cause mortality at 90 and 365 days. Secondary aims included describing the clinical spectrum, management patterns, outcomes, and seasonal distribution of mucormycosis, comparing histopathologic diagnosis with other routinely available diagnostic modalities, and describing the species distribution of culture-confirmed cases.

## Materials and methods

### Study design and setting

This was a multicenter, retrospective cohort study conducted across 27 tertiary-care centers in Türkiye. The protocol was approved by the Istanbul Medipol University Non-Interventional Clinical Research Ethics Committee (Approval No. 1203; 28 November 2024) and conformed to the Declaration of Helsinki.

### Patient selection and case definition

We included adults (≥ 18 years) with proven mucormycosis diagnosed between 1 January 2004 and 31 December 2024. The date of diagnosis was defined as the date of the first microbiological or histopathological evidence consistent with mucormycosis. Proven invasive fungal disease was defined according to the 2020 European Organization for Research and Treatment of Cancer (EORTC)/Mycoses Study Group–Education and Research Consortium (MSGERC) consensus as meeting at least one of the following criteria [14]:

Histopathological evidence of broad, pauciseptate hyphae with right-angle or irregular branching invading tissue, demonstrated by special stains or direct microscopy of a biopsy specimen; or Culture isolation of a Mucorales species from a normally sterile site obtained by aspiration or biopsy.

To avoid confounding by pandemic-associated cases, patients with microbiologically confirmed severe acute respiratory syndrome coronavirus 2 (SARS-CoV-2) infection within 90 days prior to mucormycosis diagnosis were excluded (*n* = 27). In accordance with the predefined multicenter protocol, patients with other proven or probable secondary or concomitant infections were also excluded to ensure that clinical outcomes could be attributed specifically to mucormycosis. The excluded infections were as follows: hospital-acquired Gram-negative pneumonia (including ventilator-associated pneumonia, VAP; *n* = 3), Aspergillus co-infection (*n* = 1), cytomegalovirus (CMV) pneumonia (*n* = 3), catheter-related bloodstream infection (*n* = 4), dental abscess with Actinomyces growth (*n* = 1), and Pneumocystis jirovecii pneumonia (PCP; *n* = 2). This exclusion criterion was defined a priori to prevent diagnostic overlap and ensure accurate attribution of outcomes to mucormycosis.

### Data collection

Investigators at each center screened laboratory, pathology, and microbiology records to identify eligible cases. Across all 27 participating tertiary-care centers, data were retrieved using a standardized, keyword-based search of consultation notes, microbiology, and pathology reports (keywords: “mucor,” “mucormycosis,” “mucorales,” “broad, nonseptate/pauciseptate hyphae” and their Turkish equivalents), followed by manual verification by site investigators. ICD code–only retrieval was avoided because of inconsistent coding in earlier years. A standardized case-report form (Microsoft Forms) captured:

- Demographics: age, sex.
- Underlying conditions and risk factors: DM, diabetic ketoacidosis (DKA), HSCT, SOT, chronic kidney disease, dialysis, iron chelation therapy, corticosteroid use, neutropenia, history of trauma (e.g., traffic accidents, earthquakes, falls, burns) or surgical procedures, and intravenous drug use.
- Clinical presentation: anatomical sites of involvement, signs and symptoms.
- Diagnostic modalities: direct microscopy, culture, histopathology, species identification.
- Antifungal therapy: agents, dosages, initiation timing, and duration.
- Surgical interventions: type of debridement or resection, the intervals from symptom onset and from diagnosis (start of antifungal therapy) to the first surgical procedure, and the total number of procedures.
- Outcomes: all-cause mortality at 90 and 365 days; time to death.

All entries were made by trained coordinators and independently adjudicated by two infectious diseases physicians before database lock. Data were abstracted for the index hospitalization and follow-up through 365 days after diagnosis. Follow-up data for one-year survival were obtained from institutional medical records, outpatient follow-up documentation, and archived hospital information systems. When available, these data were cross-checked against the national death notification registry to ensure completeness and accuracy.

Among 280 eligible patients, survival data were available for 278; two cases without complete 90-day/365-day follow-up were excluded from survival analyses but retained in baseline risk-factor assessments.

### Definition of key covariates

History of sinusitis was defined as a documented or reported diagnosis of chronic or recurrent rhinosinusitis before the index mucormycosis episode. This criterion was considered fulfilled if the patient or caregiver reported a previous diagnosis during admission history taking, an otorhinolaryngology or infectious diseases note documenting sinusitis/rhinosinusitis was present in the electronic medical record within the 12 months preceding mucormycosis, or prior imaging showed findings consistent with chronic rhinosinusitis. Acute sinonasal symptoms that were part of the index mucormycosis presentation were not coded as a “history of sinusitis.” This definition was adapted from the European Position Paper on Rhinosinusitis and Nasal Polyps [15].

Prior corticosteroid use was defined, in accordance with the 2020 EORTC/MSGERC host factor criteria, as prolonged systemic corticosteroid therapy at a dose of ≥ 0.3 mg/kg/day prednisone equivalent for ≥ 3 weeks within the 60 days preceding symptom onset [14]. This definition is consistent with the Global guideline for the diagnosis and management of mucormycosis by the ECMM/MSGERC, which identifies systemic corticosteroid exposure as a major host factor associated with mucormycosis risk [10].

Aggressive debridement was defined as surgical removal of all macroscopically involved and non-viable tissue aiming at clear margins, including one or more of the following: endoscopic or open clearance of multiple paranasal sinuses with or without resection of adjacent structures (e.g., palate, orbital exenteration, partial/total maxillectomy, skull-base drilling), lobectomy or pulmonary segmentectomy for pulmonary disease, or wide/radical excision for cutaneous disease. These procedures could be performed as single or staged interventions. This definition reflects the recommendations of the ECMM/MSGERC Global Guideline for Mucormycosis and prior surgical series emphasizing early and extensive source control [6, 10, 11, 12].

Limited debridement was defined as conservative endoscopic sinus surgery or focal removal of necrotic tissue confined to the primarily involved site (e.g., single-sinus functional endoscopic sinus surgery, focal palatal debridement, or limited cutaneous excision) without removal of adjacent bone or orbital structures. This category corresponds to conservative procedures occasionally reported in multicenter mucormycosis cohorts when extensive surgery was contraindicated [16, 17].

Recent chemotherapy was defined as any systemic cytotoxic or targeted antineoplastic regimen administered within 30 days before the onset of mucormycosis-related symptoms, as documented in oncology or hematology records. This timeframe aligns with recognized risk windows for invasive mold diseases in hematology and transplant populations [10, 14].

### Outcomes

The primary outcomes were all-cause mortality at 90 and 365 days after diagnosis. Secondary outcomes included the clinical spectrum, management patterns, outcomes, and seasonal distribution of mucormycosis; the diagnostic performance of histopathology, culture, and direct microscopy; real-world patterns of antifungal therapy and surgical management.

For time-to-event survival analyses, follow-up time was measured from the date of mucormycosis diagnosis to death or last documented clinical encounter. Right-censoring was applied at prespecified administrative cutoffs: patients who were alive at day 90 or day 365 were censored on those days. Two patients whose 1-year vital status could not be verified were excluded a priori from survival analyses; therefore, all 278 patients included in the Kaplan–Meier and Cox models had ≥ 365 days of potential follow-up, and no individuals were censored before day 365 due to insufficient follow-up.

### Statistical analysis

Continuous variables are summarized as median [interquartile range, IQR] or mean ± standard deviation (SD).and compared using Student’s t-test or the Mann–Whitney U test, as appropriate. Categorical variables are presented as counts (percentages) and compared using the χ² test or Fisher’s exact test.

Overall survival was estimated by the Kaplan–Meier (KM) method and compared using the log-rank test. Because KM estimates account for censoring, KM-based survival probabilities may show small discrepancies compared with crude proportions reported in descriptive tables. Cox proportional-hazards models were used to identify predictors of 90- and 365-day mortality. Variables with *p* < 0.10 in univariable analyses, together with clinically relevant covariates, were entered into multivariable models. Proportional-hazards assumptions were assessed using Schoenfeld residuals. To mitigate immortal time bias, surgical intervention was modeled as a time-dependent covariate.

Missing data for continuous variables were imputed using the cohort mean when approximately normally distributed and the median otherwise. A complete-case sensitivity analysis yielded similar results. Two-sided p values < 0.05 were considered statistically significant. Analyses were performed using R version 4.2.1 (R Foundation for Statistical Computing, Vienna, Austria) with the packages survival, survminer, and broom.

## Results

### Study characteristics

A total of 280 adults with proven mucormycosis were included. The median age at diagnosis was 60 years [49–69], and 156 (56.1%) were male. A prior history of mucormycosis was documented in 22 patients (7.9%), while 76 (27.3%) had a history of sinusitis.

The most frequent underlying comorbidity was DM (197; 70.9%), of which 54 patients (19.4%) presented with DKA. Hematological malignancy was the second most common condition (75; 27.0%). Within this group, acute myeloid leukemia was predominant (31; 39.7%), followed by acute lymphoblastic leukemia (16; 20.5%) and lymphoma (11; 14.1%).

HSCT had been performed in 13 patients (4.7%), of whom 11 (84.6%) underwent allogeneic transplantation. The median interval from HSCT to symptom onset was 90 days [20–240]. SOT was recorded in 7 patients (2.5%), with a mean interval from SOT to symptom onset of 206.7 ± 139.4 days.

Trauma or surgery within the preceding 90 days occurred in 29 patients (10.4%), with a median interval to symptom onset of 10.0 days [IQR, 7.0–20.0]. A history of chemotherapy was noted in 68 patients (24.5%), with a median interval from last chemotherapy to symptoms of 12.0 days [IQR, 7.0–18.0] (Table 1).

**Table 1:** Impact of epidemiological factors and medical history on clinical outcomes in patients with mucormycosis

Variables	All Patients (*n* = 280)	90–Day			365–Day		
		Death (*n* = 137)	Recovery (*n* = 141)	*p* Value	Death (*n* = 153)	Recovery (*n* = 125)	*P* Value
Age (years)	60.0 [49.0–69.0]	58.0 [46.0–69.0]	62.0 [51.0–69.0]	0.088^1^	58 [46–69]	63 [51–69]	0.090^1^
Gender (male)	156 (56.1%)	82 (59.9%)	74 (52.5%)	0.216^2^	90 (58.8%)	66 (52.8%)	0.314^2^
Previous history of mucormycosis	22 (7.9%)	10 (7.3%)	12 (8.5%)	0.708^2^	11 (7.2%)	11 (8.8%)	0.621^2^
Diabetes mellitus	197 (70.9%)	100 (73%)	97 (68.8%)	0.441^2^	111 (72.5%)	86 (68.8%)	0.494^2^
Diabetic ketoacidosis at diagnosis	54 (19.4%)	31 (22.6%)	23 (16.3%)	0.183^2^	32 (20.9%)	22 (17.6%)	0.487^2^
HIV^a^ infection	2 (0.7%)	1 (0.7%)	1 (0.7%)	1.000^3^	2 (1.3%)	0 (0%)	0.503^3^
Hypertension	73 (26.3%)	34 (24.8%)	39 (27.7%)	0.590^2^	39 (25.5%)	34 (27.2%)	0.747^2^
Chronic kidney failure	66 (23.7%)	32 (23.4%)	34 (24.1%)	0.882^2^	34 (22.2%)	32 (25.6%)	0.510^2^
Dialysis	24 (8.6%)	12 (8.8%)	12 (8.5%)	0.941^2^	12 (7.8%)	12 (9.6%)	0.604^2^
COPD^b^	9 (3.2%)	5 (3.6%)	4 (2.8%)	0.747^3^	6 (3.9%)	3 (2.4%)	0.521^3^
Decompensated cirrhosis	9 (3.2%)	3 (2.2%)	6 (4.3%)	0.501^3^	3 (2%)	6 (4.8%)	0.307^3^
Solid cancer	9 (3.2%)	5 (3.6%)	4 (2.8%)	0.747^3^	7 (4.6%)	2 (1.6%)	0.193^3^
Rheumatological disease	10 (3.6%)	5 (3.6%)	5 (3.5%)	1.000^3^	5 (3.3%)	5 (4%)	0.758^3^
History of sinusitis	76 (27.3%)	23 (16.8%)	53 (37.6%)	**< 0.001** ^**2**^	24 (15.7%)	52 (41.6%)	**< 0.001** ^**2**^
Hematological malignancy	75 (27%)	35 (25.5%)	40 (28.4%)	0.596^2^	38 (24.8%)	37 (29.6%)	0.373^2^
Hematological Malignancy Subtype				0.332^3^			0.161^3^
AML^c^	31 (39.7%)	12 (8.8%)	19 (13.5%)		14 (9.2%)	17 (13.6%)	
ALL^d^	16 (20.5%)	10 (7.3%)	6 (4.3%)		12 (7.8%)	4 (3.2%)	
Lymphoma	11 (14.1%)	3 (2.2%)	8 (5.7%)		3 (2%)	8 (6.4%)	
Aplastic anemia	6 (7.7%)	4 (2.9%)	2 (1.4%)		4 (2.6%)	2 (1.6%)	
MM^e^	4 (5.1%)	3 (2.2%)	1 (0.7%)		3 (2%)	1 (0.8%)	
CLL^f^	4 (5.1%)	2 (1.5%)	2 (1.4%)		2 (1.3%)	2 (1.6%)	
MDS^g^	4 (5.1%)	1 (0.7%)	3 (2.1%)		1 (0.7%)	3 (2.4%)	
CML^h^	1 (1.3%)	1 (0.7%)	0 (0%)		1 (0.7%)	0 (0%)	
NK^i^ cell plasmacytoma	1 (1.3%)	0 (0%)	1 (0.7%)		0 (0%)	1 (0.8%)	
HSCT^j^	13 (4.7%)	8 (5.8%)	5 (3.5%)	0.365^2^	9 (5.9%)	4 (3.2%)	0.292^2^
Type of Stem Cell Transplantation				0.487^3^			1.000^3^
Autologous HSCT	2 (15.4%)	2 (1.5%)	0 (0%)		2 (1.3%)	0 (0%)	
Allogeneic HSCT	11 (84.6%)	6 (4.4%)	5 (3.5%)		7 (4.6%)	4 (3.2%)	
Days from HSCT to symptom onset	90.0[20.0–240.0]	67.5[27.0–262.8]	90.0[20.0–240.0]	1.00^1^	45 [18–210]	165[72.5–244.25]	0.486^1^
Neutropenia (≥ 14 days)	44 (15.8%)	21 (15.3%)	23 (16.3%)	0.822^2^	24 (15.7%)	20 (16%)	0.943^2^
Neutropenia days at fungal symptom onset	12.0 [5.0–21.0]	15.0 [10.0–21.0]	10.0 [5.0–18.0]	0.106^1^	16 [10–21]	8.5[5–15.75]	**0.038** ^**1**^
SOT^k^	7 (2.5%)	3 (2.2%)	4 (2.8%)	1.000^3^	3 (2%)	4 (3.2%)	0.704^3^
Days from SOT to symptom onset	206.7 ± 139.4	262.3 ± 165.0	165.0 ± 123.7	0.445^4^	350 [211–357.5]	150 [60–255]	0.445^4^
Antifungal prophylaxis history	37 (13.3%)	19 (13.9%)	18 (12.8%)	0.787^2^	22 (14.4%)	15 (12%)	0.561^2^
Antifungal Prophylaxis Regimen				0.600^3^			0.084^3^
Fluconazole	7 (18.9%)	3 (2.2%)	4 (2.8%)		3 (2%)	4 (3.2%)	
Posaconazole	12 (32.4%)	5 (3.6%)	7 (5%)		5 (3.3%)	7 (5.6%)	
Voriconazole	18 (48.6%)	11 (8%)	7 (5%)		14 (9.2%)	4 (3.2%)	
Trauma or surgery in last 90 days	29 (10.4%)	14 (10.2%)	15 (10.6%)	0.909^2^	14 (9.2%)	15 (12%)	0.439^2^
Day from trauma or surgery to complaints	10.0 [7.0–20.0]	8.5 [6.0–17.5]	15.0 [8.0–20.5]	0.204^1^	8.5 [6–17.5]	15 [8–20.5]	0.197^1^
Chemotherapy history	68 (24.5%)	25 (18.2%)	43 (30.5%)	**0.017** ^**2**^	26 (17%)	42 (33.6%)	**0.001** ^**2**^
Last Chemotherapy before symptoms (days)	12.0 [7.0–18.0]	14.0 [7.0–20.0]	12.0 [7.0–17.0]	0.818^1^	14 [8–18]	10 [7–14]	0.172^1^
History of deferoxamine use	9 (3.2%)	7 (5.1%)	2 (1.4%)	0.099^3^	3 (2%)	6 (4.8%)	0.307^3^
History of steroid use	80 (28.8%)	41 (29.9%)	39 (27.7%)	0.676^2^	53 (34.6%)	27 (21.6%)	**0.017** ^**2**^
Cumulative steroid dose	655.0 [200.0–976.0]	600.0 [210.0–1147.0]	670.0 [200.0–895.0]	0.870^1^	640 [200–950]	716 [388.5–1205]	0.258^1^
History of statin use	40 (14.4%)	18 (13.1%)	22 (15.6%)	0.558^2^	20 (13.1%)	20 (16%)	0.489^2^
At the time of diagnosis the patient				0.900^2^			0.679^2^
Inpatient	166 (59.7%)	82 (59.9%)	84 (59.6%)		90 (58.8%)	76 (60.8%)	
Outpatient	71 (25.5%)	36 (26.3%)	35 (24.8%)		42 (27.5%)	29 (23.2%)	
ICU^l^	41 (14.7%)	19 (13.9%)	22 (15.6%)		21 (13.7%)	20 (16%)	

1 Mann–Whitney U, 2 Chi-square test, 3 Fisher’s exact test, 4 t-test, mean ± SD for normally distributed continuous variables; median (Q1–Q3) for non-normally distributed continuous variables; n (%) for categorical variables.

^*a*^*HIV* human immunodeficiency virus, ^b^*COPD* chronic obstructive pulmonary disease, ^*c*^*AML* acute myeloid leukemia, ^*d*^*ALL* acute lymphoblastic leukemia, ^e^*MM* multiple myeloma, ^*f*^*CLL* chronic lymphocytic leukemia, ^g^*MDS* myelodysplastic syndrome, ^h^*CML* Chronic Myeloid Leukemia ^i^*NK* Natural Killer, ^j^*HSCT* hematopoietic stem cell transplantation, ^k^*SOT* solid organ transplantation, ^l^*ICU* Intensive Care Unit.

### Clinical presentation and diagnostics

#### Clinical forms and seasonality

**Fig. 1:**
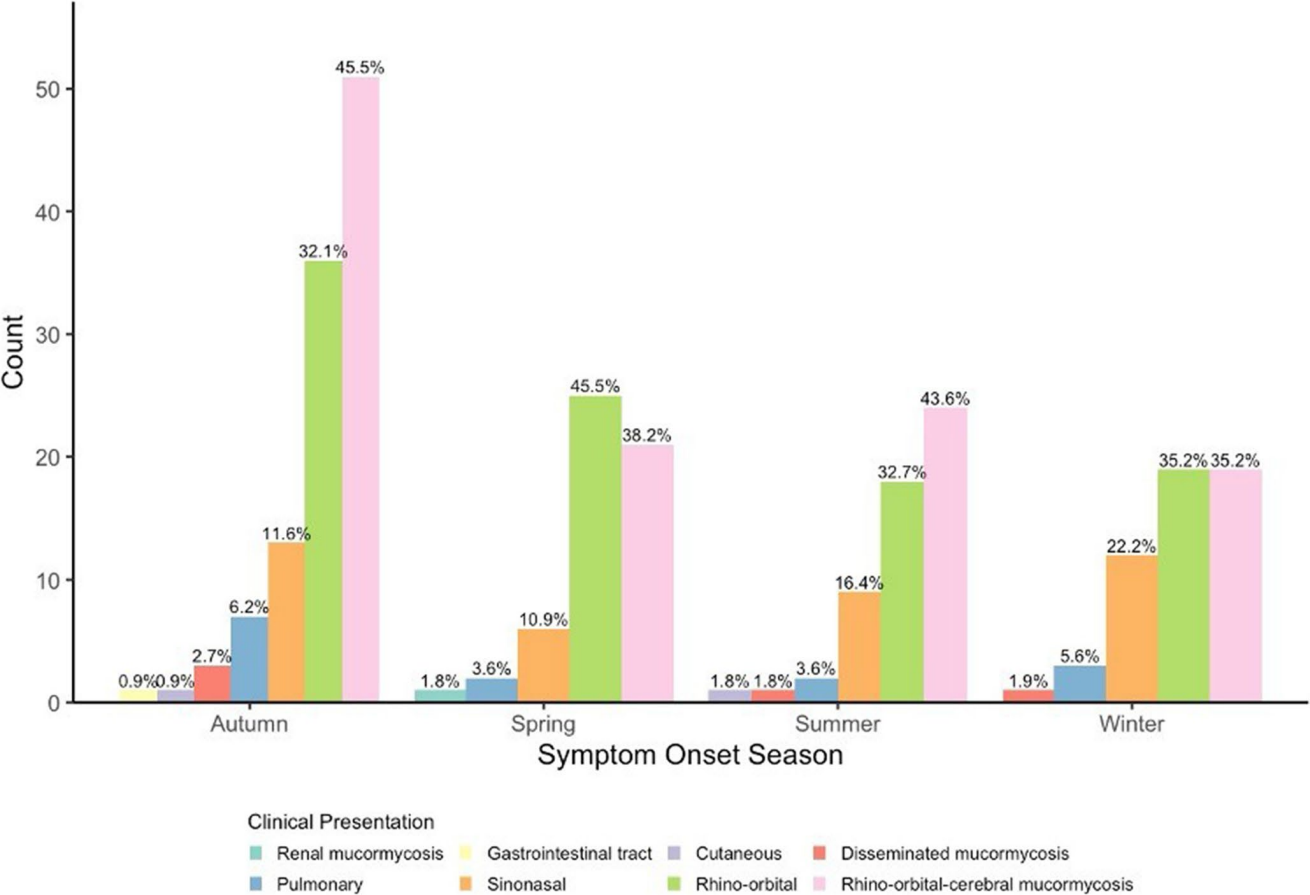
Seasonal distribution of mucormycosis by clinical presentation. The most common clinical presentation was rhino-orbital-cerebral mucormycosis (115; 41.4%), followed by rhino-orbital disease (99; 35.6%) and sinonasal involvement (40; 14.4%). Symptom onset was most frequent in autumn (113; 40.6%), with equal proportions observed in winter, spring, and summer (each 55; 19.8%). Rhino-orbital and rhino-orbital-cerebral forms predominated across all seasons, whereas sinonasal cases were more frequent in winter, and pulmonary involvement peaked in autumn

The most frequent clinical presentation was ROCM (115; 41.4%), followed by rhino-orbital disease (99; 35.6%) and sinonasal (40; 14.4%). Symptom onset occurred most often in autumn (113; 40.6%), with similar proportions across winter, spring, and summer (each 55; 19.8%). ROCM and rhino-orbital disease predominated across all seasons, particularly in autumn and winter, whereas sinonasal disease was more common in winter, and pulmonary cases peaked in autumn (Fig. 1).

#### Presenting symptoms

**Fig. 2:**
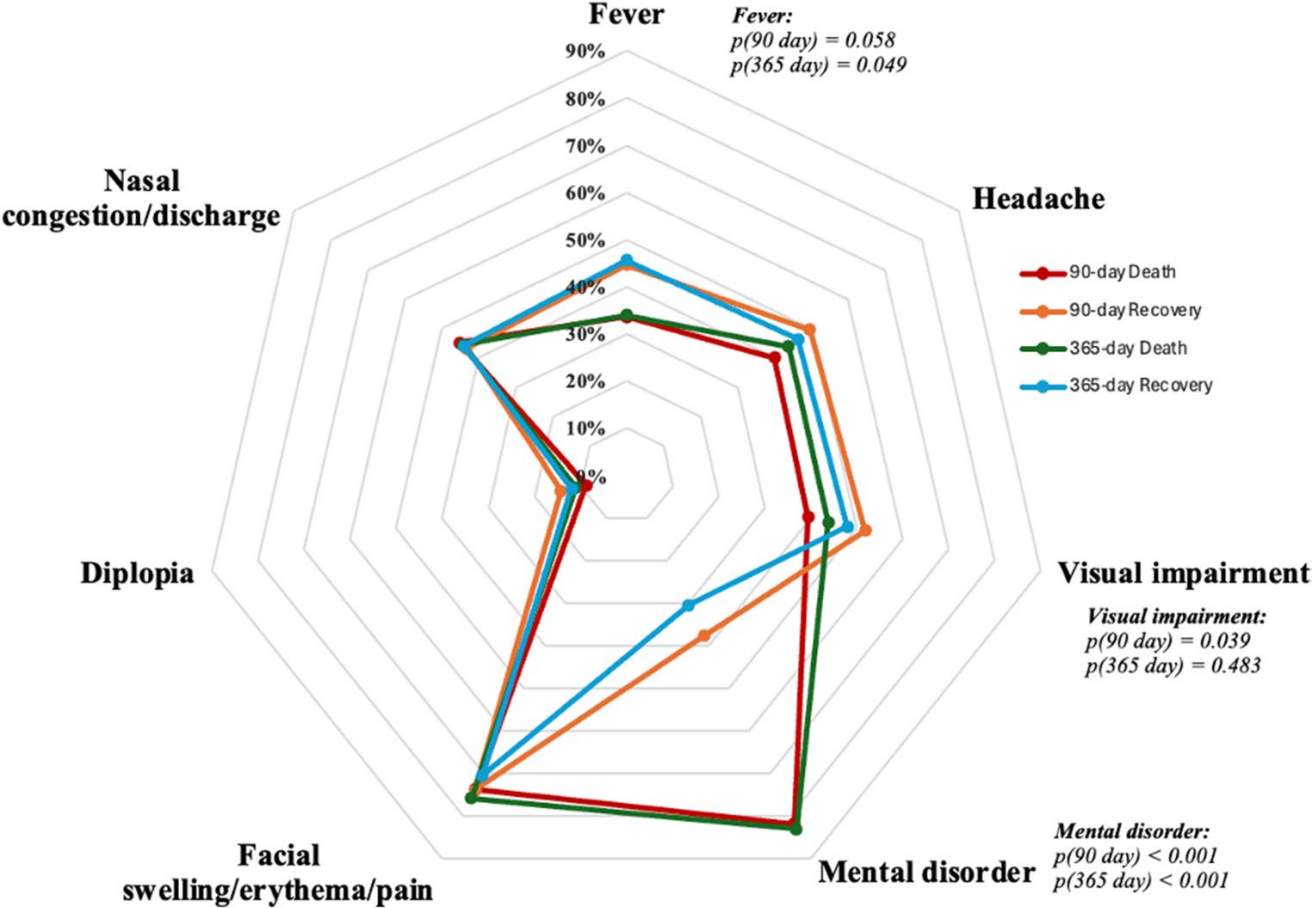
Distribution of presenting symptoms in patients with mucormycosis. This radar plot depicts the distribution of presenting symptoms at diagnosis, stratified by clinical outcomes at 90 and 365 days in patients with mucormycosis. Values represent the proportion of patients within each outcome group exhibiting the respective symptom at presentation. Statistical comparisons between death and recovery groups at each time point were performed using univariate analysis, with corresponding *p*-values displayed. Fever demonstrated a significant association with 365-day mortality (*p* = 0.049) but not with 90-day mortality (*p* = 0.058). Visual impairment was significantly associated with 90-day mortality (*p* = 0.039) but not with 365-day mortality (*p* = 0.483). Mental disorder was strongly associated with mortality at both 90 and 365 days (both *p* < 0.001)

At presentation, the most frequent symptoms were facial swelling, erythema, or pain (73.7%), altered mental status (59.4%), and visual impairment (45.7%). Other manifestations such as headache, nasal congestion or discharge, fever, and diplopia were less common (Fig. 2).

#### Physical examination findings

**Fig. 3:**
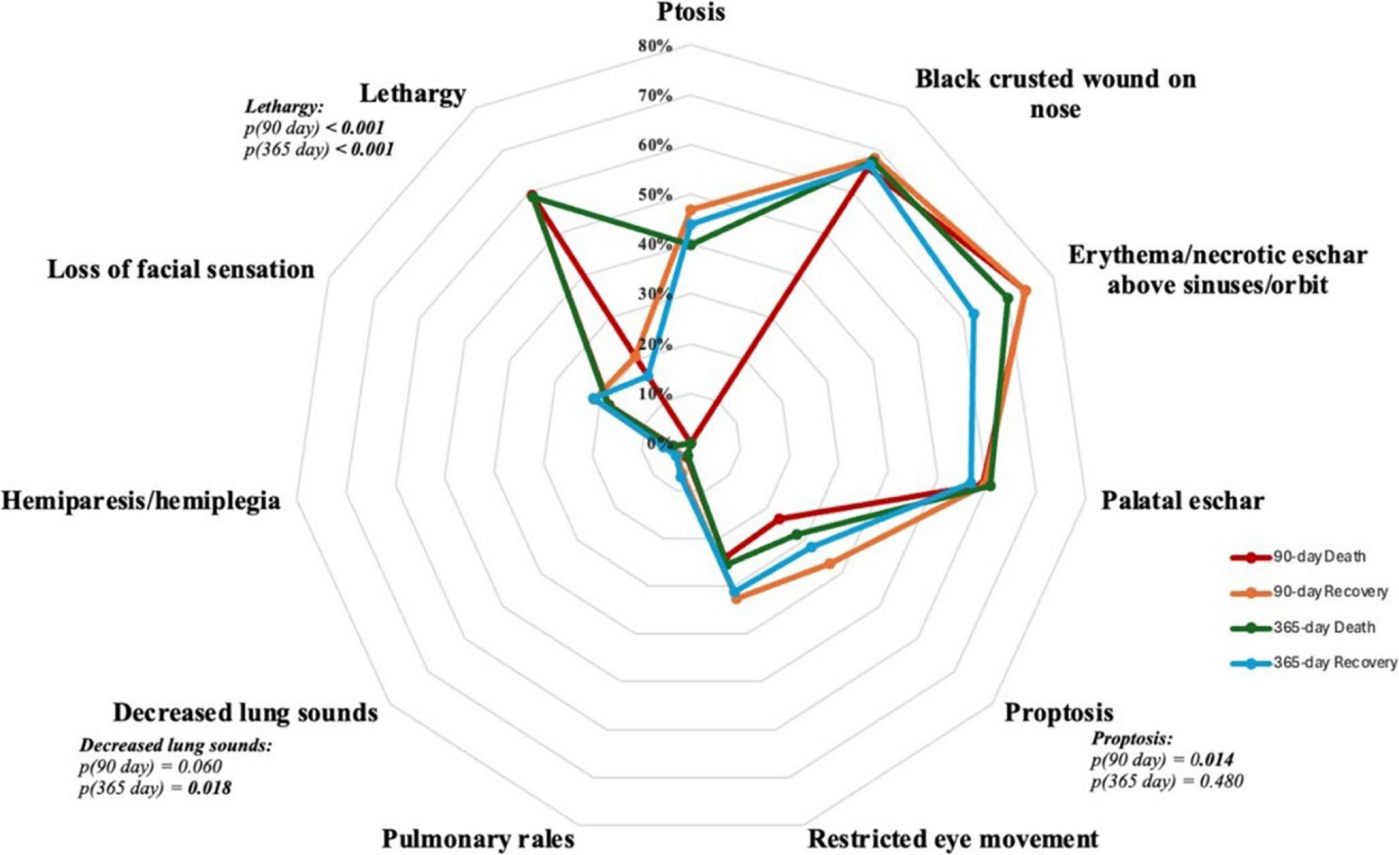
Distribution of physical examination findings in patients with mucormycosis. This radar plot depicts the distribution of physical examination findings at diagnosis, stratified by clinical outcomes at 90 and 365 days in patients with mucormycosis. Values represent the proportion of patients within each outcome group exhibiting the respective finding at presentation. Statistical comparisons between death and recovery groups at each time point were performed using univariate analysis, with corresponding *p*-values displayed. Lethargy was strongly associated with mortality at both 90 and 365 days (both *p* < 0.001). Proptosis was significantly associated with 90-day mortality (*p* = 0.014) but not with 365-day mortality (*p* = 0.480). Decreased lung sounds were significantly associated with 365-day mortality (*p* = 0.018) but not with 90-day mortality (*p* = 0.060)

The most frequent physical findings were erythema over the sinuses/orbit (73.7%), necrotic eschar in the nasal cavity (66.9%), and palatal eschar (59.4%) (Fig. 3).

#### Diagnostic methods and pathogen identification

Histopathology confirmed the diagnosis in 234 patients (84.2%). Microbiological evidence was obtained by stained microscopy alone in 67 patients (23.9%), culture alone in 58 (20.7%), and by both methods in 58 (20.7%). Among culture-positive cases, the most frequently isolated species were *Rhizomucor pusillus* (47; 40.2%), *Mucor* circinelloides (37; 31.6%), and *Rhizopus arrhizus* (32; 27.4%) (Table 2).

**Table 2:** Impact of laboratory parameters laboratory parameters and treatment strategies on survival outcomes in patients with mucormycosis

	All Patients (*n* = 280)	90–Day			365–Day		
		Death (*n* = 137))	Recovery (*n* = 141)	*p* value^1^	Death (*n* = 153)	Recovery (*n* = 125)	*p* Value^1^
^a^WBC **(**cells/µL**)**	10245.0 [4125.0–17450.0]	10885.0 [2980.0–17877.5]	9930.0 [5752.5–17012.5]	0.999	10,800 [2660–17870]	10,100 [6300–17050]	0.783
^b^PMNL **(**cells/µL**)**	7490.0 [2400.0–14400.0]	7600.0 [1582.5–14945.0]	7200.0 [3400.0–12640.0]	0.962	7490 [1500–14810]	7545 [4002.5–12980]	0.699
^c^Lym **(**cells/µL**)**	940.0 [470.0–1570.0]	725.0 [360.0–1177.5]	1115.0 [600.0–1790.0]	0.001	720 [330–1200]	1117.5 [647.5–1900]	< 0.001
^d^CRP (mg/L)	126.0 [46.0–200.0]	139.0 [67.0–236.0]	114.5 [34.8–185.5]	0.013	141 [69–235]	98.5 [34–176.5]	0.001
^e^PCT (ng/mL)	0.9 [0.2–4.5]	1.0 [0.3–6.5]	0.6 [0.2–2.0]	0.110	0.905 [0.2475–5.5]	0.57 [0.2–3.75]	0.285
Serum Iron (µg/dL)	12.0 [5.0–32.0]	17.0 [6.5–36.5]	12.0 [4.2–27.5]	0.190	15 [6–35]	12 [4.75–28]	0.362
Ferritin (ng/mL)	840.0 [338.0–1660.5]	963.0 [339.0–2000.0]	813.0 [340.0–1129.0]	0.193	1028 [371–2000]	780 [332.75–1108.25]	0.121
^f^ESR (mm/h)	59.0 [36.0–85.0]	56.0 [30.0–82.8]	59.0 [38.5–87.5]	0.364	62.5 [30.25–84.75]	58 [37.5–86]	0.764
Species identified				**< 0.001** ^**1**^			0.380^1^
*Rhizopus arrhizus*	32 (27.4%)	2 (1.5%)	30 (21.3%)		18 (11.8%)	14 (11.2%)	
*Rhizomucor pusillus*	47 (40.2%)	43 (31.4%)	4 (2.8%)		26 (17%)	21 (16.8%)	
*Lichtheimia corymbifera*	1 (0.9%)	1 (0.7%)	0 (0%)		1 (0.7%)	0 (0%)	
*Mucor circinelloides*	37 (31.6%)	19 (13.9%)	18 (12.8%)		26 (17%)	11 (8.8%)	
Symptom to antifungal start (days)	5.0 [2.0–8.0]	5.0 [2.0–8.0]	5.0 [2.0–8.0]	0.896^*3*^	5 [2–8]	5 [2–8]	0.666^*3*^
Surgical operation				**0.003** ^**2**^			**< 0.001** ^**2**^
Aggressive debridement	95 (34.2%)	47 (34.3%)	48 (34%)		56 (36.6%)	39 (31.2%)	
Limited debridement	134 (48.2%)	76 (55.5%)	58 (41.1%)		82 (53.6%)	52 (41.6%)	
No debridement	49 (17.6%)	14 (10.2%)	35 (24.8%)		15 (9.8%)	34 (27.2%)	
Symptom to surgery interval (days)	6.0 [4.0–10.0]	6.0 [3.0–10.0]	7.0 [5.0–10.0]	0.103^*3*^	6 [3–10]	7 [4–10]	0.090^*3*^
Number of surgical operations	2.0 [1.0–3.0]	2.0 [1.0–3.0]	1.0 [1.0–2.0]	0.061^*3*^	2 [1–3]	1 [1–2]	**0.005** ^***3***^
Initial antifungal regimen				0.345^1^			0.603^1^
Liposomal amphotericin B	240 (87.3%)	118 (86.1%)	122 (86.5%)		131 (85.6%)	109 (87.2%)	
Liposomal amphotericin B + Caspofungin	4 (1.5%)	2 (1.5%)	2 (1.4%)		2 (1.3%)	2 (1.6%)	
Liposomal amphotericin B + Posaconazole	23 (8.4%)	13 (9.5%)	10 (7.1%)		15 (9.8%)	8 (6.4%)	
Posaconazole	4 (1.5%)	0 (0%)	4 (2.8%)		0 (0%)	4 (2.8%)	
Amphotericin B deoxycholate	1 (0.4%)	0 (0%)	1 (0.7%)		1 (0.4%)	0 (0%)	
Amphotericin B dosage (mg/kg/day)	5.0 [5.0–5.0]	5.0 [5.0–5.0]	5.0 [5.0–5.0]	0.682^*3*^	5 [5–5.5]	5 [5–5]	0.669^*3*^
Hyperbaric oxygen therapy	11 (4%)	4 (2.9%)	7 (5%)	0.382^2^	5 (3.3%)	6 (4.8%)	0.550^2^
Days in ICU^g^ (days)	7.0 [3.0–12.0]	7.0 [3.0–13.0]	7.0 [3.0–11.0]	0.541^*3*^	7 [3–12.5]	7 [3–11.5]	0.757^*3*^
Mechanical ventilation	121 (43.5%)	101 (73.7%)	20 (14.2%)	**< 0.001** ^**2**^	106 (69.3%)	15 (12%)	**< 0.001** ^**2**^

^1^Fisher’s exact test,^2^ Chi-square test, ^3^Mann–Whitney U, median (Q1–Q3) for continuous variables; n (%) for categorical variables.

^a^*WBC* white blood cell, ^b^*PMNL* polymorphonuclear leukocyte, ^c^*Lym* lymphocyte, ^d^*CRP* C-reactive protein, ^e^*PCT* procalcitonin, ^f^*ESR* erythrocyte sedimentation rate, ^g^*ICU* days in intensive care unit

### Treatment modalities

The median time from symptom onset to initiation of antifungal therapy was 5.0 days [2.0–8.0]. Liposomal amphotericin B was the predominant first-line agent (240; 87.3%). Combination regimens included liposomal amphotericin B with posaconazole (23; 8.4%) or caspofungin (4; 1.5%). Posaconazole monotherapy was used in 4 patients (1.5%), while amphotericin B deoxycholate was given in a single patient (0.4%). The median daily dose of liposomal amphotericin B was 5.0 mg/kg [5.0–5.0] (Table 2).

Surgical intervention was performed as aggressive debridement in 95 patients (34.2%) and as limited debridement in 134 patients (48.2%), whereas 49 patients (17.6%) did not undergo any surgical procedure (Table 2). The median interval from diagnosis of mucormycosis (defined as the date of initiation of antifungal therapy) to the first surgical procedure was 0 days [0–2], and the median number of operations per patient was 2 [1, 3].

The median total duration of antifungal therapy was 28 days [15.0–53.0]. The median length of intensive care unit (ICU) stay was 7.0 days [3.0–12.0], and 121 patients (43.5%) required mechanical ventilation (Table 2).

### Risk factors for mortality

#### Univariable Analysis

In univariable analyses, several demographic, clinical, and laboratory variables showed significant differences between survivors and non-survivors at both 90 and 365 days. A history of sinusitis at presentation was more frequent among survivors (90-day: 37.6% vs. 16.8%, *p* = 0.0001; 365-day: 41.6% vs. 15.7%, *p* < 0.001). Recent chemotherapy exposure was also more common in survivors (90-day: 30.5% vs. 18.2%, *p* = 0.017; 365-day: 33.6% vs. 17.0%, *p* = 0.001). Prior systemic corticosteroid use was more frequent among non-survivors in the 365-day analysis (34.6% vs. 21.6%, *p* = 0.017). Moreover, a longer duration of neutropenia at symptom onset was observed in non-survivors (median 16 vs. 8.5 days, *p* = 0.038) (Tables 1 and 2). In the subgroup of patients with diabetes mellitus, the presence of diabetic ketoacidosis was not significantly associated with mortality (90-day: 56.4% vs. 48.3%, *p* = 0.27; 365-day: 58.2% vs. 55.2%, *p* = 0.63).

In terms of clinical presentation, altered mental status was more frequent among patients who died, occurring in 81.8% of those who died within 90 days compared with 37.6% of survivors (*p* < 0.001), and in 83.0% versus 30.4% at one year (*p* < 0.001). Lethargy was also significantly more common in non-survivors (90-day: 59.1% vs. 20.6%, *p* < 0.001; 365-day: 58.8% vs. 16.0%, *p* < 0.001). At 90 days, visual impairment (51.8% vs. 39.4%, *p* = 0.039) and proptosis (36.9% vs. 23.4%, *p* = 0.014) were likewise more frequent among non-survivors (Figs. 2 and 3).

Laboratory parameters that differed significantly included lower lymphocyte counts (median 725 vs. 1115 cells/µL at 90 days, *p* = 0.001; 720 vs. 1117.5 cells/µL at 365 days, *p* < 0.001) and higher C-reactive protein concentrations (median 139 vs. 114.5 mg/L at 90 days, *p* = 0.013; 141 vs. 98.5 mg/L at 365 days, *p* = 0.001) among non-survivors (Tables 1 and 2).

Treatment-related differences were also notable The absence of surgical intervention was more frequent among survivors than non-survivors (90-day: 24.8% vs. 10.2%, *p* = 0.003; 365-day: 27.2% vs. 9.8%, *p* < 0.001). The need for mechanical ventilation was also significantly more common among non-survivors (90-day: 73.7% vs. 14.2%; 365-day: 69.3% vs. 12.0%; *p* < 0.001 for both) (Tables 1 and 2).

### Survival analysis

#### Clinical presentation

**Fig. 4:**
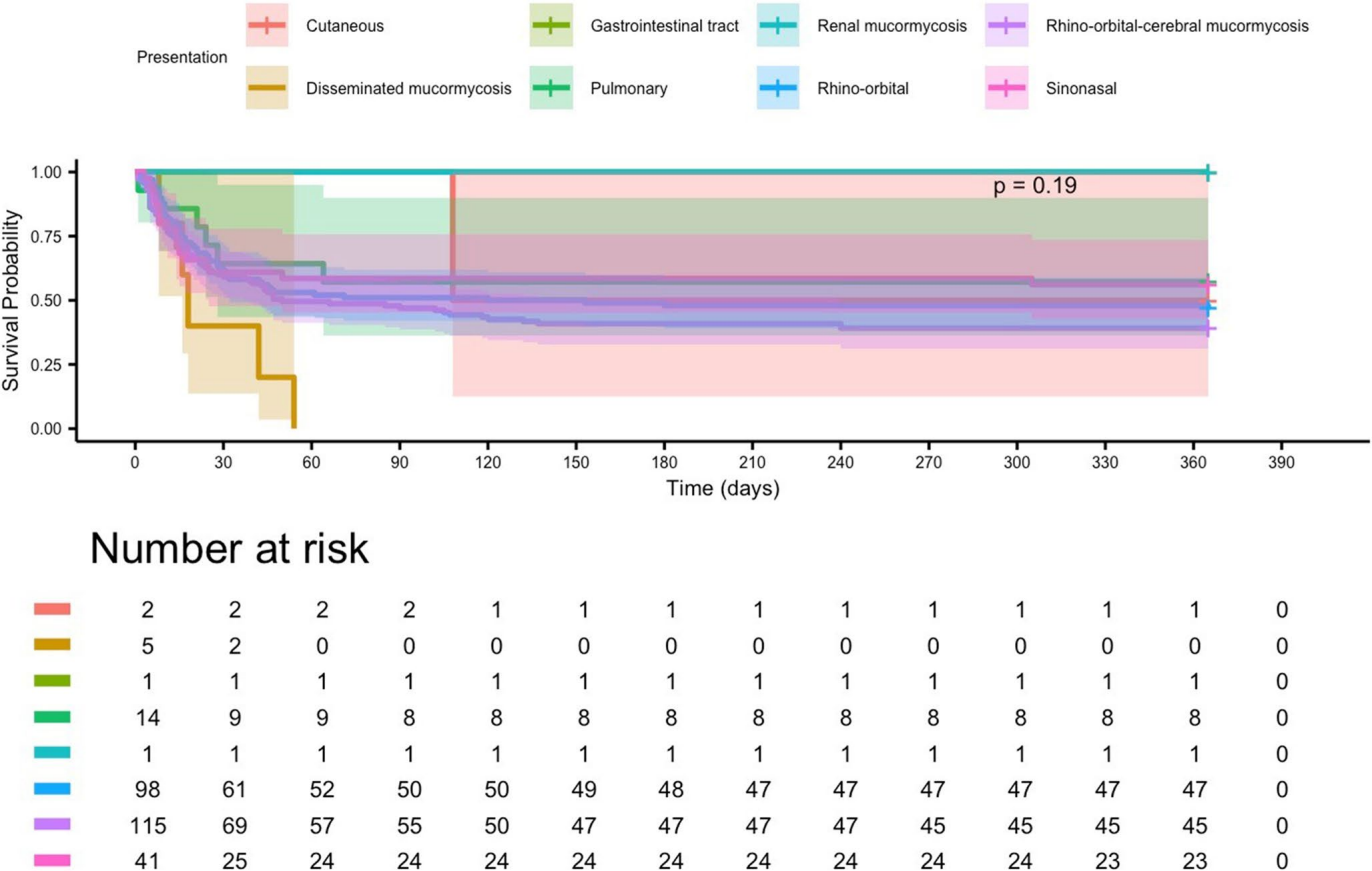
Kaplan–Meier estimates of 365-day survival stratified by clinical presentation in patients with mucormycosis. No statistically significant differences were observed between subgroups (log-rank *p* = 0.19). Disseminated mucormycosis demonstrated the lowest survival probability, with the Kaplan–Meier curve declining to approximately 25% by around day 60, whereas gastrointestinal, renal, pulmonary, and rhino-orbital presentations showed comparatively higher survival probabilities throughout follow-up

In Kaplan–Meier analysis, overall survival did not differ significantly according to the anatomic site of infection (log-rank *p* = 0.19). Among patients with sufficient sample sizes, pulmonary and rhino-orbital-cerebral mucormycosis showed comparable survival trajectories, whereas disseminated forms exhibited the poorest outcomes, with survival dropping steeply within the first month. Gastrointestinal and renal involvement were rare but associated with relatively favorable survival, although these findings should be interpreted cautiously due to limited case numbers (Fig. 4).

#### Culture results

**Fig. 5:**
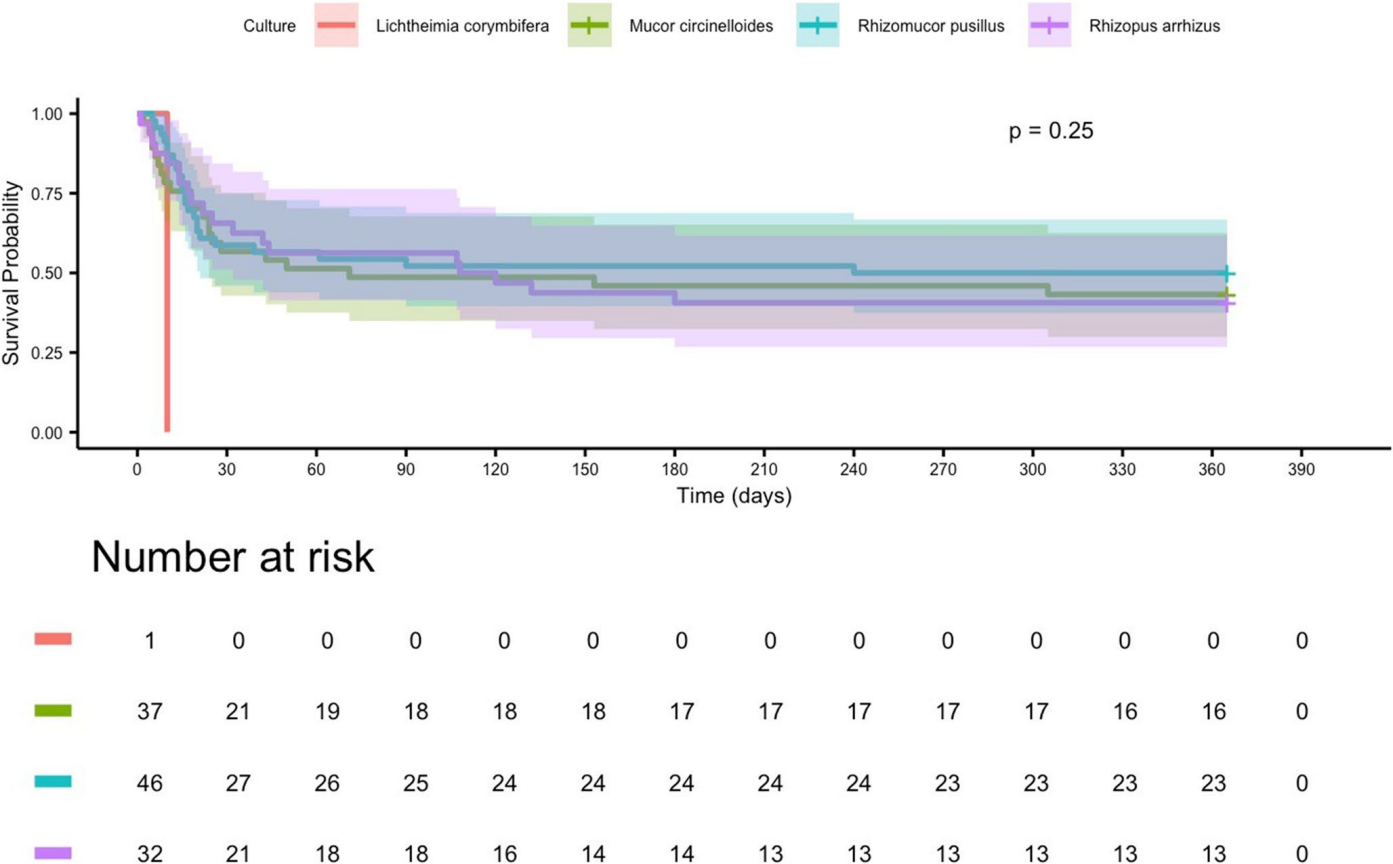
Kaplan–Meier estimates of 365-day survival stratified by Mucorales species in patients with mucormycosis. No statistically significant differences were observed between groups (log-rank *p* = 0.25). Survival probability declined rapidly within the first 30 days for all species, after which curves stabilized and remained largely parallel. *Rhizomucor pusillus* demonstrated the highest survival probability throughout follow-up, whereas *Mucor circinelloides* and *Rhizopus arrhizus* exhibited similar trajectories. The single *Lichtheimia corymbifera* case was associated with early mortality

Kaplan–Meier analysis stratified by isolated Mucorales species showed no statistically significant differences in 365-day survival (log-rank *p* = 0.25). Median survival was 120 days for patients with *R. pusillus* infection, 95 days for *M. circinelloides*, and 88 days for *R. arrhizus*. The single case of *L. corymbifera* resulted in death within 15 days (Fig. 5).

#### Amphotericin B dosage

**Fig. 6:**
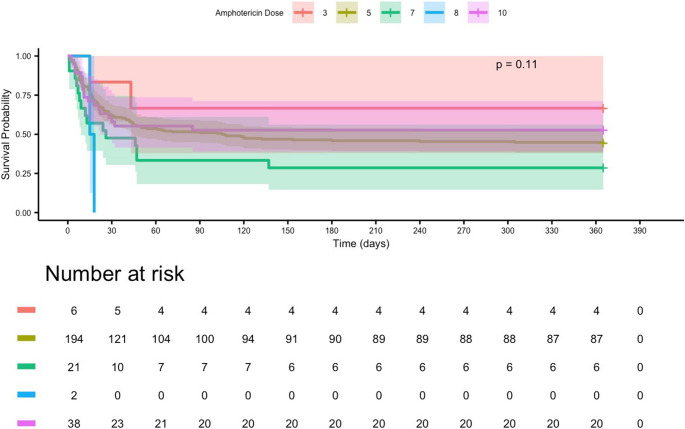
Kaplan–Meier estimates of 365-day survival stratified by initial amphotericin B dose in patients with mucormycosis. No statistically significant differences were observed between dosing groups (log-rank *p* = 0.11). Patients receiving higher initial doses (≥ 8 mg/kg) appeared to have marginally better long-term survival probabilities compared with those receiving lower doses (≤ 5 mg/kg), although these trends did not reach statistical significance. Very low (3 mg/kg) and very high (10 mg/kg) dose groups were small in size, limiting statistical power for definitive comparisons

Kaplan–Meier analysis stratified by initial liposomal amphotericin B dose showed no statistically significant differences in 365-day survival (log-rank *p* = 0.11). Median survival was 118 days in patients receiving higher cumulative doses (≥ 8 g) and 94 days in those receiving lower doses (≤ 5 g). Minimal-dose (3 g) and very high-dose (10 g) subgroups had median survivals of 71 and 102 days, respectively, but each accounted for less than 5% of the cohort (Fig. 6).

#### Surgical intervention

**Fig. 7:**
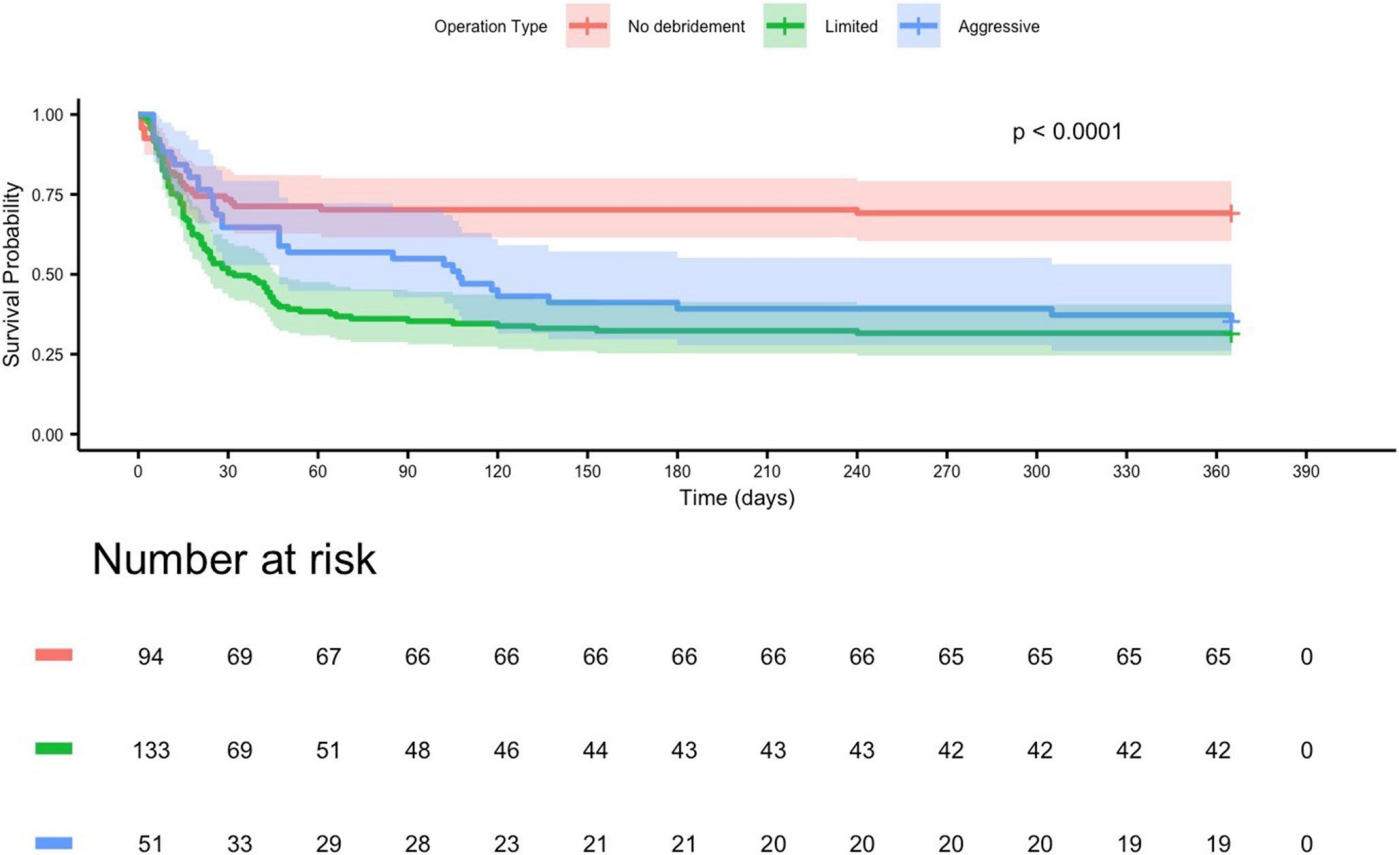
Kaplan–Meier estimates of 365-day survival stratified by surgical intervention in patients with mucormycosis. Crude Kaplan–Meier survival curves differed significantly between groups (log-rank *p* < 0.0001). Patients who did not undergo debridement showed the highest crude survival probabilities, whereas those who received surgical intervention—either limited or aggressive debridement—had lower crude survival. Among operated patients, aggressive debridement appeared to be associated with slightly higher crude survival compared with limited debridement. These differences, however, are descriptive and do not account for disease severity at presentation or the timing of surgery. Because surgery is typically offered to patients with more extensive or rapidly progressive disease, confounding by indication and immortal-time bias may influence these crude estimates. In multivariable Cox models incorporating surgery as a time-dependent covariate, surgical intervention was not independently associated with long-term mortality, indicating that the apparent paradox in the crude curves likely reflects baseline disease severity rather than a harmful effect of surgery

Kaplan–Meier analysis demonstrated significant differences in 365-day survival across surgical groups (log-rank *p* < 0.001). Median survival was 72 days for patients undergoing aggressive debridement and 54 days for those receiving limited debridement. In contrast, patients who did not undergo surgery had a numerically longer median survival of 192 days (Fig. 7).

#### Initial care setting

**Fig. 8:**
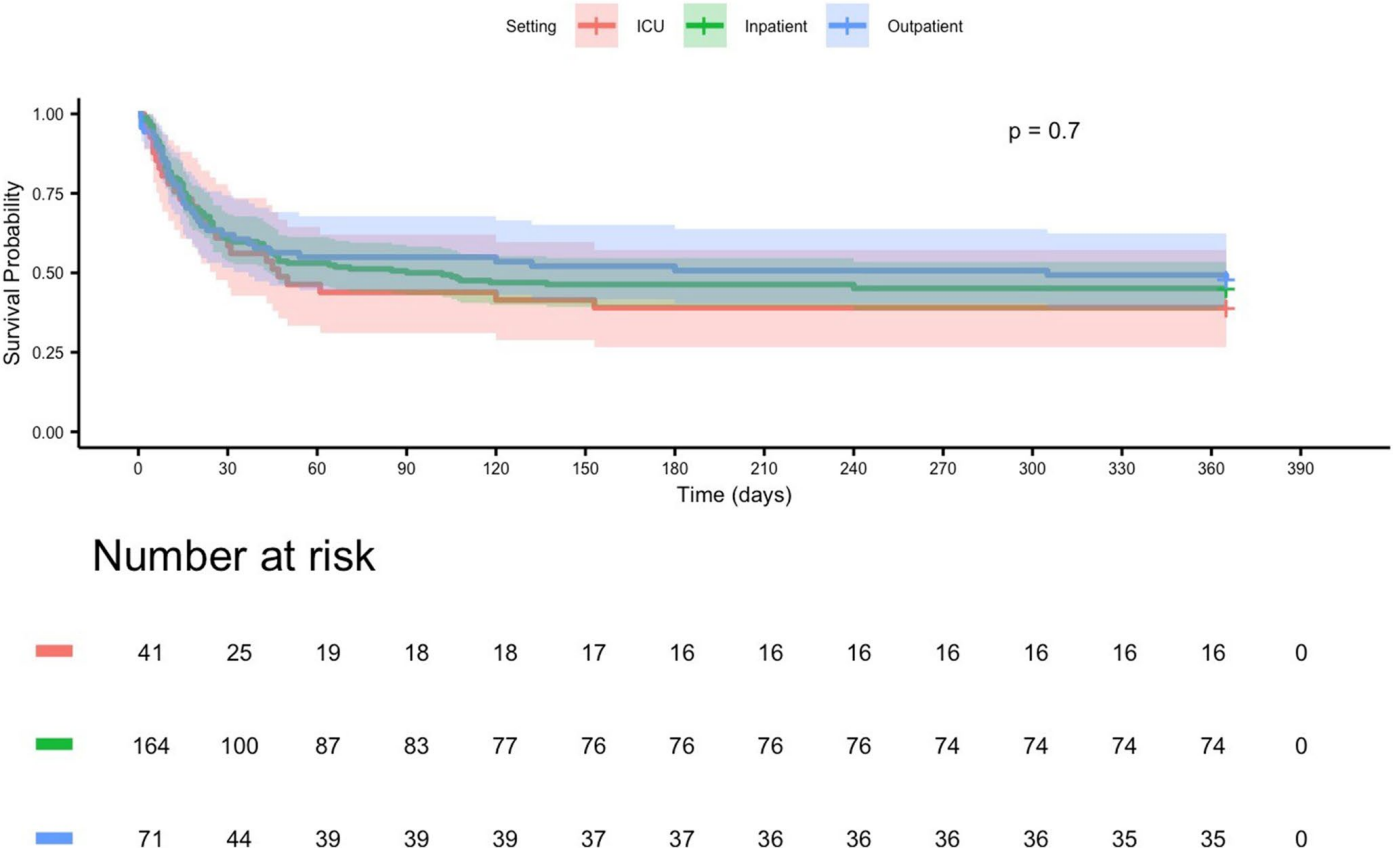
Kaplan–Meier estimates of 365-day survival stratified by initial care setting in patients with mucormycosis. No statistically significant differences in survival were observed between patients initially managed in the Intensive Care Unit (ICU), inpatient ward, or outpatient setting (log-rank *p* = 0.70). Survival curves for all three groups remained largely overlapping throughout follow-up, indicating comparable long-term outcomes regardless of the initial treatment location

Kaplan–Meier analysis stratified by initial care setting revealed no statistically significant differences in 365-day survival (log-rank *p* = 0.70). Median survival was 86 days for ICU patients, 124 days for inpatients, and 136 days for outpatients (Fig. 8).

### Multivariable cox regression analysis

Cox regression analyses were conducted separately for 90-day and 365-day mortality. Younger age showed a borderline protective effect against 90-day mortality (*p* = 0.056) and was significantly protective at 365 days (HR = 0.99; 95% CI: 0.98–1.00; *p* = 0.037). Because the proportional hazards assumption was violated, surgical intervention was modeled with a time interaction term in the 90-day analysis. This revealed that surgery increased the hazard of death within the first 90 days (HR = 1.02; 95% CI: 1.01–1.03; *p* = 0.003), whereas its effect was not statistically significant at 365 days (*p* = 0.190). The discriminative performance of the 90-day model was moderate (concordance = 0.598; SE = 0.024), exceeding that of the 365-day model (concordance = 0.564; SE = 0.023). Global significance tests for the 365-day model were not statistically significant (all *p* = 0.50), whereas the 90-day model approached statistical significance across likelihood ratio (*p* = 0.07), Wald (*p* = 0.10), and log-rank (*p* = 0.08) (Table 3).

**Table 3:** Cox regression analysis of factors associated with 90-day and 365-day mortality

		90-Days Mortality		365-Days Mortality		
Variable		HR (95% CI)	*p*-Value	HR (95% CI)	*p*-Value	Reference Category
Age		0.99 (0.98–1.98)	0.056	**0.99 (0.98–1.98)**	**0.037**	Per 1-year increase
Sex	Male	0.91 (0.64–1.3)	0.618	0.97 (0.69–1.35)	0.839	Female
Diabetic ketoacidosis at diagnosis	Yes	0.89 (0.57–1.39)	0.618	0.93 (0.61–1.42)	0.738	No
Hematological malignancy	Yes	0.84 (0.48–1.45)	0.525	0.85 (0.51–1.44)	0.552	No
Surgical intervention (time-dependent)		**1.02 (1.01–1.03)**	**0.003**	**1 (0.99–1.99)**	**0.190**	No
Neutropenia days at fungal symptom onset	< 14 days	0.69 (0.29–1.61)	0.387	0.73 (0.34–1.57)	0.417	No
	³14 days	0.84 (0.38–1.86)	0.669	1.05 (0.5–2.19)	0.901	No
Antifungal prophylaxis history	Yes	1.32 (0.69–2.55)	0.403	1.12 (0.62–2.03)	0.698	No
Recent trauma/surgery (time-dependent)	Yes	1.01 (0.99–1.03)	0.336	0.91 (0.53–1.56)	0.723	No
Chemotherapy history	Yes	0.84 (0.55–1.3)	0.440	0.87 (0.59–1.3)	0.502	No
Lethargy	Yes	1.2 (0.83–1.74)	0.338	1.22 (0.86–1.75)	0.267	No
Mechanical ventilation	Yes	1.08 (0.75–1.55)	0.684	1.15 (0.81–1.62)	0.436	No
Lymphocyte		1 (1–1)	0.392	1 (1–1)	0.314	Per 1-unit increase
Symptom to antifungal start days		1 (0.97–1.04)	0.865	1 (0.97–1.04)	0.870	Per 1-day increase

*Due to the violation of the proportional hazards assumption, the ‘Trauma or surgery last 90 days’ variable was modeled with a time-varying coefficient (with a *time interaction) in the 90-day mortality model.

In the 365-day mortality model, as this variable did not violate the assumption, it was included with a fixed coefficient.“90-day model: Concordance = 0.598 (se = 0.024). Overall significance tests: Likelihood ratio test (p = 0.07), Wald test (p = 0.1), Score (logrank) test (p = 0.08). 365-day model: Concordance = 0.564 (se = 0.023). Overall significance tests: Likelihood ratio test (p = 0.5), Wald test (p = 0.5), Score (logrank) test (p = 0.5).

## Discussion

### Key findings

In this nationwide multicenter cohort of 280 adults with proven mucormycosis from 27 tertiary centers in Türkiye over a 20-year period, overall mortality remained substantial, with approximately 42% of patients dying within 90 days and about half by one year despite widespread use of liposomal amphotericin B and frequent surgical management. Restricting the analysis to proven cases according to the 2020 EORTC/MSGERC criteria and excluding SARS-CoV-2–associated infections likely contributed to a more homogeneous, non-pandemic cohort and reduced the risk of diagnostic misclassification.

Diabetes mellitus, often complicated by ketoacidosis, was the predominant underlying condition, followed by hematological malignancy. Taken together, this predominance of diabetes and hematological malignancy highlights a dual burden of metabolic and immunosuppressive risk factors in this middle-income setting.

In Kaplan–Meier analyses, survival differed across surgical groups, but lower crude survival among surgically treated patients likely reflected more advanced baseline disease in those selected for surgery rather than a detrimental effect of surgery itself. Overall, these findings provide a contemporary clinical overview of mucormycosis and underscore the need for timely diagnosis and standardized multidisciplinary care pathways.

### Epidemiology and risk factors

The median age in this cohort was 60 years, and 56.1% were male, consistent with large European multicenter studies (median ages 55–62 years; male 52–60%) 9], but younger than Indian series, which often exceed 65–70% [3]. DM was present in 70.9% of patients, markedly higher than the 20–40% reported in European cohorts [4] and consistent with the 54–76% described in India, where poor glycemic control is prevalent [2]. This reflects the high burden of inadequately controlled DM in our setting and reinforces its critical role as a modifiable risk factor. Hematological malignancy was observed in 27.0% of patients, lower than the 40–50% reported in high-income countries [10] but consistent with middle-income settings dominated by metabolic comorbidities.

### Clinical spectrum and seasonality

The most frequent presentation was ROCM (41.4%), followed by rhino-orbital (35.6%) and sinonasal disease (14.4%). In multicenter studies, ROCM usually accounts for 60–70% of cases, pulmonary disease for 10–15%, and disseminated disease for 5–6% [16]. Higher ROCM rates in India (> 75%) are linked to uncontrolled DM [2, 3], whereas European cohorts report lower ROCM proportions with more pulmonary and disseminated cases [4]. Our classification separated rhino-orbital and sinonasal forms from ROCM, contributing to a lower ROCM proportion. Symptom onset peaked in autumn (40.6%), with less pronounced seasonal variation otherwise. Seasonal patterns have been inconsistently described; Indian studies report monsoon-related peaks [2, 3], whereas European cohorts show less distinct trends [4]. The relatively modest seasonal variation in our study likely reflects the large proportion of immunocompromised patients in whom host factors outweigh environmental exposure.

### Clinical manifestations

The most common symptoms were facial swelling, erythema, or pain (73.7%), altered mental status (59.4%), and visual impairment (45.7%). On examination, necrotic eschar over the sinuses/orbit (73.7%), black crusted nasal wounds (66.9%), and palatal eschar (59.4%) were frequent. These hallmark signs of Mucorales angioinvasion are consistent with prior reports [3, 7]. Neurological findings, especially altered mental status, were strongly associated with mortality, exceeding frequencies reported in French (42%) [4] and Indian (55–60%) [3] cohorts, suggesting delayed recognition or advanced disease at presentation. Similar seasonal variations have also been reported in Eastern Mediterranean cohorts [17, 18], supporting the regional relevance of our findings. The relatively modest seasonal variation in this study likely reflects the large proportion of immunocompromised patients in whom host factors outweigh environmental exposure.

### Treatment patterns

The median time from symptom onset to antifungal initiation was 5 days, which was longer than reported in French cohorts (2–3 days) [4] and above the ≤ 3-day threshold recommended by ECMM/MSGERC [10]. Liposomal amphotericin B was the predominant first-line agent (87.3%), consistent with international practice [3, 4]. Combination therapy (9.9%) was used selectively in refractory or disseminated disease [11]. Surgical intervention was performed in 82.4% of patients (34.2% aggressive, 48.2% limited debridement), comparable to other multicenter series [2, 4]. Although crude Kaplan–Meier curves suggested poorer survival among surgically treated patients compared with those without surgery, this finding should be interpreted with caution because surgery was more frequently offered to patients with more severe baseline disease.

### Mortality and prognostic indicators

In this cohort, all-cause mortality was 42.1% at 90 days and 49.3% at 365 days, within the 40–60% range of prior multicenter studies [3, 4]. Disseminated disease had the highest fatality (87.5%), followed by gastrointestinal involvement (83.3%), consistent with prior reports [7, 9]. Localized ROCM (38.0%) and isolated sinonasal disease (30.0%) had comparatively better survival, aligning with patterns described in India and Europe [3, 4].

Notably, a history of sinusitis was associated with improved outcomes, likely reflecting earlier recognition, while corticosteroid exposure and prolonged neutropenia predicted poorer survival. Neurological symptoms, especially altered mental status, were powerful adverse prognostic factors, highlighting the need for early recognition.

In this cohort, crude Kaplan–Meier curves stratified by surgical status appeared to show better survival in patients who did not undergo surgery than in those who underwent aggressive or limited debridement. This counterintuitive pattern is likely explained by confounding by indication and immortal-time bias, because surgery was generally reserved for patients with more extensive or rapidly progressive disease and was performed after diagnosis rather than at time zero. When surgery was modeled as a time-dependent covariate in the Cox regression, it was not independently associated with long-term mortality. Accordingly, the survival estimates in Fig. 7 should be viewed as descriptive; they are not directly comparable with the presentation-based survival probabilities in Fig. 4 and should not be interpreted as evidence that withholding surgery improves prognosis.

### COVID-19 (coronavirus disease 2019)–associated mucormycosis (CAPM)

Mortality in CAPM is often reported at 50–70%, and in some series approaches 80% at 12 weeks [19], which is notably higher than the 42% and 49% observed at 90 and 365 days in our cohort. Such differences may be related to diagnostic heterogeneity, treatment delays during the pandemic, and the predominance of pulmonary disease in CAPM. By focusing on proven, non-COVID cases, our study offers a clearer reference point for mucormycosis outcomes outside the pandemic context.

### Strengths of the study

This study represents a nationwide multicenter cohort of adult patients with proven mucormycosis collected over two decades from 27 tertiary-care centers in Türkiye. Strict case definitions based on the 2020 EORTC/MSGERC criteria were applied to reduce misclassification, and SARS-CoV-2–related or other concomitant infections were deliberately excluded to limit pandemic-era diagnostic heterogeneity. To our knowledge, this study provides one of the most comprehensive and systematically verified multicenter datasets on proven mucormycosis from a middle-income country, thereby strengthening the validity of long-term outcome assessments.

### Limitations

This study has several limitations. First, its retrospective design and reliance on existing medical records may have led to underascertainment of some clinical variables, and non-uniform electronic medical record systems across centers limited retrieval of early cases and detailed temporal trend analyses. Second, our 2004–2024 study period spans substantial changes in diagnostic modalities and in antifungal and surgical management of mucormycosis, which may have introduced temporal heterogeneity in case ascertainment and outcomes that could not be fully accounted for in our analyses. Third, referral bias is possible, as tertiary centers are more likely to capture advanced or complicated cases, and the absence of a mucormycosis-specific standardized severity score hindered stratification by baseline disease severity and cross-study comparisons. Fourth, incomplete antifungal susceptibility testing restricted our ability to comprehensively assess resistance patterns. Finally, although we adjusted for key clinical covariates, residual confounding by baseline disease severity cannot be ruled out when interpreting the association between surgical timing and outcomes.

## Conclusions

This nationwide, multicenter study of 280 adults with proven mucormycosis over two decades demonstrates that the disease continues to carry an unacceptably high mortality, with 42% of patients dying within 90 days and nearly half within one year despite guideline-adherent antifungal therapy and frequent surgical intervention. DM emerged as the predominant underlying risk factor, underscoring the impact of the growing metabolic disease burden in middle-income countries. Taken together, these results highlight the urgent need to strengthen surveillance systems, establish standardized severity assessment tools, and optimize treatment strategies tailored to local epidemiology in order to improve outcomes in this devastating infection.

## Supplementary Information

Below is the link to the electronic supplementary material.

Supplementary File 1 (PDF 314 KB)

## Data Availability

The data that support the findings of this study are not publicly available due to ethical restrictions. Data may be made available from the corresponding author upon reasonable request and following additional approval from the Institutional Ethics Committee.
