# Supplementary material for: Epidemiological profile and clinical outcomes of patients with mucormycosis: the multicenter retromucor study from Türkiye (2004–2024)
Source: Eur J Clin Microbiol Infect Dis. 2026 Jan 23;45(2):537–55. doi: 10.1007/s10096-025-05397-x (PMC12987872; doi:10.1007/s10096-025-05397-x)
Supplement: Supplementary file 1 — (PDF 314 KB) [file 10096_2025_5397_MOESM1_ESM.pdf]

T.C.
İSTANBUL MEDİPOL ÜNİVERSİTESİ
Girişimsel Olmayan Klinik Araştırmalar Etik Kurulu Başkanlığı

Sayı : E-10840098-202.3.02-7590 10/12/2024
Konu : Etik Kurulu Kararı

Sayın Yeliz Çiçek

Üniversitemiz Girişimsel Olmayan Klinik Araştırmalar Etik Kuruluna yapmış olduğunuz ‘Mukormikozis Tanılı Olguların Risk Faktörleri ve Klinik Sonuçlarının Kapsamlı Analizi: Retromukor Çalışması (2004-2024)’ isimli başvurunuz Etik Kurulumuzca değerlendirilerek uygun görülmüş olup Etik Kurulu kararı ekte sunulmuştur.

Bilgilerinize rica ederim.

Dr. Öğr. Üyesi Mahmut TOKAÇ
Girişimsel Olmayan Klinik Araştırmalar
Etik Kurulu Başkanı

Bu belge, güvenli elektronik imza ile imzalanmıştır.
Evrağınızı https://turkiye.gov.tr/istanbul-medipol-universitesi-ebys linkinden 4BC4925FX8 kodu ile dogrulayabilirsiniz.

İSTANBUL MEDİPOL ÜNİVERSİTESİ
GİRİŞİMSEL OLMAYAN KLİNİK ARAŞTIRMALAR
ETİK KURULU KARAR FORMU

**BAŞVURU BİLGİLERİ**					
	**ARAŞTIRMANIN AÇIK ADI**	Mukormikozis Tanılı Olguların Risk Faktörleri ve Klinik Sonuçlarının Kapsamlı Analizi: Retromukor Çalışması (2004-2024)			
	**KOORDİNATÖR/SORUMLU ARAŞTIRMACI UNVANI/ADI/SOYADI**	Yeliz Çiçek			
	**KOORDİNATÖR/SORUMLU ARAŞTIRMACININ UZMANLIK ALANI**	Enfeksiyon Hastalıkları ve Klinik Mikrobiyoloji			
	**KOORDİNATÖR/SORUMLU ARAŞTIRMACININ BULUNDUĞU MERKEZ**	İstanbul			
	**DESTEKLEYİCİ**	-			
	**ARAŞTIRMAYA KATILAN MERKEZLER**	TEK MERKEZ ☐	ÇOK MERKEZ ☒	ULUSAL ☒	ULUSLARARASI ☐

**Değerlendirilen Belgeler**	**Belge Adı**	**Tarihi**	**Versiyon Numarası**	**Dili**
	ARAŞTIRMA PROTOKOLÜ/PLANI			Türkçe ☐ İngilizce ☐ Diğer ☐
	OLGU RAPOR FORMU			Türkçe ☐ İngilizce ☐ Diğer ☐
	BİLGİLENDİRİLMİŞ GÖNÜLLÜ OLUR FORMU			Türkçe ☐ İngilizce ☐ Diğer ☐
**Karar Bilgileri**	**Karar No: 1203**	**Tarih: 28.11.2024**		
	Yukarıda bilgileri verilen Girişimsel Olmayan Klinik Araştırmalar Etik Kurulu başvuru dosyası ile ilgili belgeler araştırmanın gerekçe, amaç, yaklaşım ve yöntemleri dikkate alınarak incelenmiş ve araştırmanın etik ve bilimsel yönden uygun olduğuna **“oybirliği”** ile karar verilmiştir.			

Bu belge, güvenli elektronik imza ile imzalanmıştır.
Evrağınızı https://turkiye.gov.tr/istanbul-medipol-universitesi-ebys linkinden 4BC4925FX8 kodu ile dogrulayabilirsiniz.

İSTANBUL MEDİPOL ÜNİVERSİTESİ
GİRİŞİMSEL OLMAYAN KLİNİK ARAŞTIRMALAR
ETİK KURULU KARAR FORMU

**İSTANBUL MEDİPOL ÜNİVERSİTESİ GİRİŞİMSEL OLMAYAN KLİNİK ARAŞTIRMALAR ETİK KURULU**	
**BAŞKANIN UNVANI / ADI / SOYADI**	Dr. Öğr. Üyesi Mahmut TOKAÇ

**Unvanı/Adı/Soyadı**	**Uzmanlık Alanı**	**Kurumu**	**Cinsiyet**		**Araştırma ile ilişki**		**Katılım ***		**İmza**
			E	K	E	H	E	H	
Dr. Öğr. Üyesi Mahmut TOKAÇ	Tıp Tarihi ve Etik	İstanbul Medipol Üniversitesi	☒	☐	☐	☒	☒	☐	E imzalıdır
Prof. Dr. Mete ÜNGÖR	Endodonti	İstanbul Medipol Üniversitesi	☒	☐	☐	☒	☒	☐	E imzalıdır
Prof. Dr. İlknur KESKİN	Histoloji ve Embriyoloji	İstanbul Medipol Üniversitesi	☐	☒	☐	☒	☒	☐	E imzalıdır
Doç. Dr. Devrim TARAKCI	Fizyoterapi ve Rehabilitasyon	İstanbul Medipol Üniversitesi	☒	☐	☐	☒	☒	☐	E imzalıdır
Dr. Öğr. Üyesi Neziha HACIHASANOĞLU ÇAKMAK	Biyokimya	İstanbul Medipol Üniversitesi	☐	☒	☐	☒	☒	☐	E imzalıdır
Dr. Öğr. Üyesi Erman GEDİKLİ	Sağlık Yönetimi	İstanbul Medipol Üniversitesi	☒	☐	☐	☒	☒	☐	E imzalıdır
Dr. Öğr. Üyesi Pakize YİĞİT	Biyoistatistik/ Sayısal Yöntemler	İstanbul Medipol Üniversitesi	☐	☒	☐	☒	☒	☐	E imzalıdır

* :Toplantıda Bulunma

Girişimsel Olmayan Klinik Araştırmalar
Etik Kurulu Sekreteri
Esra KAN

Bu belge, güvenli elektronik imza ile imzalanmıştır.
Evrağınızı https://turkiye.gov.tr/istanbul-medipol-universitesi-ebys linkinden 4BC4925FX8 kodu ile dogrulayabilirsiniz.
